# Effects of high-intensity resistance training within concurrent training programs on athletic performance in elite athletes: a systematic review

**DOI:** 10.7717/peerj.21807

**Published:** 2026-10-09

**Authors:** Tao Wang, Yee Guan Ng, Saidon Amri, Tengku Fadilah Kamalden, Zhendong Gao, Qian Yuan

**Affiliations:** 1 Department of Sport Studies, Faculty of Educational Studies, Universiti Putra Malaysia, Serdang, Selangor, Malaysia; 2 Department of Environmental and Occupational Health, Faculty of Medicine and Health Sciences, Universiti Putra Malaysia, Serdang, Selangor, Malaysia; 3 National Sports Complex, Bukit Jalil, Kuala Lumpur, Malaysia; 4 Department of Physical Education, Northwest University, Xi’an, Shaanxi, China; 5 School of Athletic Performance, Shanghai University of Sport, Shanghai, China

**Keywords:** High-intensity resistance training, Elite athletes, Athletic performance, Muscular strength, Power, Endurance, Concurrent training, Speed

## Abstract

**Objectives:**

This systematic review aimed to evaluate the effects of high-intensity resistance training (HIRT), operationally defined as resistance protocols utilizing loads ≥ 75% of one-repetition maximum (1RM), on athletic performance outcomes—including muscular strength, power, jumping performance, speed, preplanned change-of-direction (COD) speed, and endurance—among elite athletes.

**Methods:**

Following PRISMA 2020 guidelines, a systematic search was conducted in Web of Science, PubMed, Scopus and SPORTDiscus to identify studies that applied HIRT in elite athlete populations. Google Scholar was additionally utilized for forward and backward citation tracking. Twelve studies met the inclusion criteria and were assessed using the PEDro scale. Data on intervention characteristics, testing protocols, and performance outcomes were extracted and synthesized narratively.

**Results:**

HIRT interventions (range: 5–18 weeks) demonstrated significant improvements in maximal strength (*e.g.*, squat and bench press 1RM) and neuromuscular power-related outcomes across the majority of studies. Improvements in sprint speed, sport-specific velocity, and preplanned COD speed were also observed, although evidence for COD performance was limited to a single study. Endurance-related outcomes, including time to exhaustion, movement economy, and selected time-trial measures, generally favored HIRT, with no clear evidence of detrimental effects on aerobic-capacity-related outcomes. However, all included studies implemented HIRT within concurrent training programs rather than as an isolated intervention.

**Conclusions:**

The available evidence suggests that HIRT may be an effective training component for improving strength, power, and selected performance outcomes in elite athletes when integrated into concurrent sport-specific training programs. The strongest support was observed for maximal strength and neuromuscular power, whereas evidence for preplanned COD speed remains preliminary and requires replication. Given the concurrent training designs, the absence of a formal GRADE assessment, and the generally small sample sizes, these findings should be interpreted as likely low-to-moderate certainty evidence rather than as proof of the isolated effects of HIRT. The review protocol was prospectively registered with the International Prospective Register of Systematic Reviews (PROSPERO) on 6 January 2025 (Registration No.: CRD42025634254).

## Introduction

Elite athletes continuously seek marginal physiological and performance improvements to enhance competitive outcomes, and resistance training has emerged as an essential component of their conditioning programs (Makaruk et al., 2024). High-intensity resistance training (HIRT) is characterized by the systematic manipulation of key training variables to optimize neuromuscular adaptations (Townsend, 2022). In this review, intensity is operationally defined as the magnitude of resistance relative to an athlete’s maximal capacity (*i.e.,* percentage of one-repetition maximum (%1RM)), whereas external load refers to the absolute mass lifted (kg) (Haff & Triplett, 2015). Typically, HIRT protocols necessitate high relative intensities (≥75% 1RM) coupled with lower repetition ranges and longer rest intervals to optimize neural drive without necessarily inducing high levels of metabolic fatigue (Williams et al., 2017; Nunes et al., 2018).

Current evidence suggests that HIRT may effectively enhance athletic performance through distinct neuromuscular adaptations, including increased motor unit recruitment and rate of force development (RFD), ultimately improving force and velocity characteristics (Deschenes & Kraemer, 2002; Lecce et al., 2026). Moreover, when performed at relatively high intensities (*e.g.*, ≥75% 1RM), resistance training provides a strong mechanical stimulus for maximal strength development and neuromuscular adaptation, while the associated metabolic stress varies according to exercise selection, training volume, and rest structure (Gabriel, Kamen & Frost, 2006; de Salles et al., 2009; Currier et al., 2026). Such adaptations typically occur without significant increases in body mass, which is particularly beneficial for athletes competing in weight-class sports or precision-based sports (Østerås, Helgerud & Hoff, 2002).

Previous studies have demonstrated that traditional hypertrophy-oriented resistance training primarily targets muscular growth and local muscular endurance (Bogdanis et al., 2009). However, for elite athletes, the ability to rapidly generate maximal force and power during fast movements—such as sprinting, jumping, or rapid directional changes—is critical (Veliz et al., 2015). Despite recognized benefits, concerns persist regarding resistance training potentially impairing flexibility, agility, or sport-specific skill execution due to increased muscle stiffness or mass (Morton et al., 2011).

The effectiveness of HIRT is not confined to power- and strength-based sports. An increasing body of evidence supports its application in elite endurance populations as well. Recent evidence from concurrent strength and endurance training research indicates that adaptations may vary according to training status and sex, but also suggests that resistance-based stimuli can be integrated without universally compromising endurance-related development (Huiberts, Wüst & Van der Zwaard, 2024). In elite sport contexts, HIRT has been associated with improvements in cycling power output, movement economy, and selected endurance-related outcomes, thereby extending its relevance across a range of athletic disciplines (Støren et al., 2008; Aagaard & Andersen, 2010; Sandbakk et al., 2014). However, the current literature on HIRT in elite athletes remains fragmented, with substantial variability in intervention protocols, performance outcomes, and participant characteristics.

Thus, the purpose of this systematic review is to critically evaluate existing literature on the effects of HIRT on athletic performance in elite athletes. In this review, ‘elite athletes’ are defined as individuals who compete at national or international levels, engage in structured sport-specific training within professional or high-performance environments, and possess a minimum of five years of continuous training experience. Specifically, this review aims to synthesize current evidence regarding the impact of HIRT on maximal strength, power, preplanned change-of-direction (COD) speed, and sport-specific performance outcomes. For the purpose of this review, HIRT is employed as an overarching term encompassing training modalities performed at intensities ≥75% 1RM. Within this framework, Maximal Strength Training represents a specific sub-category utilizing near-maximal loads (≥85% 1RM) with low repetition volumes (1–5 reps) to predominantly target neural adaptations. By addressing this research gap, this review aims to provide actionable insights for coaches, athletes, and sport scientists, optimizing training prescriptions and informing future research in elite sports performance enhancement.

## Methods

### Protocol and registration

This systematic review was conducted and reported in adherence to the Preferred Reporting Items for Systematic Reviews and Meta-Analyses (PRISMA) 2020 statement (Page et al., 2021). The review protocol was prospectively registered with the International Prospective Register of Systematic Reviews (PROSPERO) on 6 January 2025 (Registration No.: CRD42025634254).

### Eligibility criteria

This systematic review employed the PICOS (Population, Intervention, Comparison, Outcomes, Study design) model to establish inclusion and exclusion criteria. Articles were included if they met the following criteria: (a) Population: The study population consisted of elite athletes, operationally defined as competitive-level athletes (irrespective of gender or age) engaging in structured, coach-supervised training within professional or national-level environments, with a minimum of five years of continuous training experience. (b) Intervention: At least one intervention arm implemented a HIRT protocol. Consistent with our operational definition, HIRT was defined as structured resistance training performed at intensities ≥75% of 1RM, utilized either as a standalone protocol or integrated into a concurrent training program. (c) Comparison: The study included a control group (active or passive) or utilized a matched-pair design allowing for between-group comparisons. The minimum intervention duration was ≥4 weeks. (d) Outcomes: The study quantitatively assessed athletic performance outcomes, including maximal strength (*e.g.*, 1RM, isokinetic peak torque), power (*e.g.*, vertical jump, peak power output or force-time characteristics), speed or preplanned change-of-direction performance (*e.g.*, sprint times, change of direction tests), or endurance (*e.g.*, VO_2_max, running economy). (e) Study Design: The review included randomized controlled trials (RCTs) and non-randomized controlled trials (CTs) published in peer-reviewed journals. (f) Language: Only full-text articles published in English were considered.

Exclusion criteria: Studies were excluded if they: (a) recruited non-elite populations (*e.g.*, novice, recreational) or clinical populations; (b) implemented resistance training interventions where the intensity was below 75% 1RM or could not be isolated (*e.g.*, unspecified load monitoring); or (c) were non-original research (*e.g.*, reviews, editorials, conference abstracts).

### Information sources and search strategy

The systematic search was conducted on 27 December 2024. The following primary electronic databases were queried: PubMed, Scopus, Web of Science, and SPORTDiscus. Additionally, Google Scholar was utilized exclusively for forward and backward citation tracking to ensure comprehensive literature coverage. The search strategy employed Boolean operators to combine keywords related to the intervention, population, and outcomes. In general, the search strategy combined terms related to resistance training, elite or competitive athletes, and athletic performance outcomes. Because database syntax differed across platforms, the complete, verbatim search strategies for all databases and search engines, including Boolean operators and database-specific syntax, are provided in Appendix 1. To identify further relevant studies, the reference lists of all included articles were manually screened. The search was restricted to full-text articles published in English, with no limitations placed on the publication date. Grey literature (*e.g.*, dissertations, conference abstracts) was screened solely to identify potential peer-reviewed publications meeting the inclusion criteria but was not included in the final synthesis.

### Study selection

This review utilized the Zotero reference management system to remove duplicates. Two authors (TW and NY) independently screened the search results based on titles and abstracts. Inter-rater reliability was assessed using Cohen’s kappa (*κ*), with a minimum acceptable threshold set a priori at *κ* ≥ 0.70. The agreement between TW and NY during this initial screening phase was excellent (*κ* = 0.86). Subsequently, two independent authors (SA and TF) conducted a full-text review of the remaining studies according to the inclusion criteria and PICOS framework. The inter-rater agreement for the full-text eligibility assessment was also high (*κ* = 0.91). Any disagreements at either stage (*e.g.*, regarding intervention type or study design) were effectively resolved through discussion and consultation with a third author (GZ). Finally, all extracted information was systematically organized using a Microsoft Excel spreadsheet (Microsoft, Redmond, WA, USA).

### Data extraction

After selecting studies, two independent reviewers (TW and NY) extracted data using a standardized form. The extracted variables included: (1) author names and publication year; (2) type of sport or athletic discipline; (3) participant characteristics (*e.g.*, sample size, gender, age, competitive level, training experience, and anthropometric data); (4) intervention protocol details (*e.g.*, training modality, intensity (%1RM), sets, duration, and frequency); (5) comparator information (*e.g.*, control group or alternative training protocols); (6) assessment methods used to evaluate performance; and (7) quantitative outcome measures, including pre- and post-intervention values, percentage changes, effect sizes (ES) where reported, and statistical significance levels (*p*-values). The extracted information was compiled into a Microsoft Excel spreadsheet (Microsoft, Redmond, WA, USA). A third reviewer (YQ) subsequently audited the data entries to ensure completeness and accuracy.

### Quality assessment

The methodological quality of the included studies was assessed using the Physiotherapy Evidence Database (PEDro) scale, a validated tool specifically designed to evaluate the internal validity and statistical reporting of randomized controlled trials (Table 1). PEDro was selected because it provides a concise and structured appraisal of internal validity and statistical reporting in controlled intervention studies, and it has been widely applied in exercise- and resistance-training-related systematic reviews, thereby facilitating transparent comparison across studies. Although alternative tools such as the Downs and Black checklist and the Cochrane Risk of Bias tool (RoB 2) are also valuable, the former is broader and less specific to trial-based exercise interventions, whereas the latter is domain-based rather than score-based and was therefore less suited to the summary grading approach adopted in the present review. The PEDro scale consists of 11 criteria. Criterion 1 relates to external validity and is excluded from the total score calculation. Criteria 2–9 assess internal validity (*e.g.*, randomization, blinding, allocation concealment), while criteria 10–11 evaluate statistical reporting. Two independent reviewers (TW and QY) assessed each study, assigning 1 point for each satisfied criterion (total score range: 0–10). Studies were stratified based on the following quality thresholds: scores of 8–10 indicated “excellent” quality; 5–7 indicated “good” quality; 3–4 indicated “moderate” quality; and <3 indicated “low” quality (Albanese et al., 2020). It should be noted, however, that in exercise interventions, particularly high-intensity resistance training, blinding of participants and therapists (criteria 5 and 6) is rarely feasible. As a result, the practical maximum achievable PEDro score for resistance training studies is often 8/10 rather than 10/10. Accordingly, although the present review retained an absolute inclusion threshold of ≥5/10 for consistency with conventional PEDro reporting, this threshold also corresponds to 5/8 achievable points (62.5%) when interpreted relative to the practical scoring ceiling in exercise-based trials. Discrepancies in scoring were resolved through consensus or consultation with a third reviewer (NY). To ensure the robustness of the evidence synthesis, a minimum quality threshold was applied: only studies scoring ≥5 points (under the predefined PEDro-based criterion) were included in the final data extraction and synthesis. Studies scoring ≤4 points were excluded from the primary analysis to mitigate potential bias. The present review did not apply a formal GRADE assessment to rate outcome-level certainty of evidence. Accordingly, the interpretation of findings was based on study-level methodological appraisal using PEDro and qualitative synthesis across outcome domains.

**Table 1:** PEDro score.

Studies	Criteria											**Score**	**Rating**
	1	2	3	4	5	6	7	8	9	10	11		
Ramos Veliz et al. (2014)	1	1	0	1	0	0	0	1	0	1	1	5	Good
Hamilton, Paton & Hopkins (2006)	1	1	0	1	0	0	0	1	0	1	1	5	Good
Silva et al. (2022)	1	1	1	1	0	0	0	1	0	1	1	6	Good
Hoff, Gran & Helgerud (2002)	1	1	0	1	0	0	0	1	0	1	1	5	Good
Hoff, Helgerud & Wisloeff (1999)	1	1	0	1	0	0	0	1	0	1	1	5	Good
Aagaard et al. (2011)	1	1	0	1	0	0	1	1	0	1	1	6	Good
Millet et al. (2002)	1	1	0	1	0	0	0	1	0	1	1	5	Good
Sunde et al. (2010)	1	1	0	1	0	0	0	1	0	1	1	5	Good
Veliz et al. (2015)	1	1	0	1	0	0	0	1	0	1	1	5	Good
Losnegard et al. (2011)	1	1	0	1	0	0	0	1	0	1	1	5	Good
Østerås, Helgerud & Hoff (2002)	1	1	0	1	0	0	0	1	0	1	1	5	Good
Vikmoen et al. (2017)	1	1	0	1	0	0	0	1	0	1	1	5	Good

**Notes.**

Item score: 1, meets criteria; 0, does not meet criteria; Criteria: 1, Eligibility criteria; 2, Random allocation; 3, Concealed allocation; 4, Baseline comparability; 5, Blind subjects; 6, Blind therapists; 7, Blind assessors; 8, Adequate Follow-up; 9, Intention to Treat Analysis; 10, Between group comparisons; 11, Point estimates and variability.

### Data synthesis

Due to the substantial heterogeneity across the included studies regarding participant characteristics (*e.g.*, specific sporting disciplines, training experience) and the diversity of outcome measures, a statistical meta-analysis was not feasible. Consequently, a narrative synthesis was conducted following the guidance from the Centre for Reviews and Dissemination (Akers, Aguiar-Ibáñez & Baba-Akbari, 2009). A comprehensive summary of study characteristics—including participant demographics, intervention protocols (duration, frequency, intensity), and key performance outcomes—is presented in Table 2. A domain-based quantitative summary of the direction and magnitude of findings is additionally presented in Table 3. For Table 3, each study was counted once within an outcome domain if it assessed at least one relevant outcome in that domain; a study contributing outcomes to more than one domain was counted once in each applicable domain. Formal between-group effect sizes could not be consistently calculated because several included studies did not report complete group means and dispersion statistics (*e.g.*, standard deviations); the synthesis therefore relied on the reported direction of findings rather than pooled between-group effect sizes.

**Table 2:** Characteristics of the studies examined in the present review.

Study	Type of athletes	Population characteristics	Interventions	Comparison	Assessments	Outcome
Ramos Veliz et al. (2014)	National water polo players	N: 27 (EG = 16, CG = 11); Sex: M; Age: 20.4 ± 5.1 yrs; TE: 8.5 ± 4.1 yrs; ht: 180.3 ± 5.9 cm; WT: 81.4 ± 8.4 kg; BF:12.4 ± 3.2%	Length: 18 weeks; Freq: 2 times/week; concurrent in-water training: 5 times/week	EG: HIRT + In-Water Training CG: In-Water Training Only	1RM (Bench Press, Full Squat), TV, CMJ, ST (20m Swim)	1RM (Bench Press): ↑10.53% (EG), ↔ (CG); 1RM (Full Squat): ↑14.21% (EG), ↔ (CG); TV: ↑2.76% (EG), ↔ (CG); CMJ: ↑6.9% (EG), ↔ (CG); ST: ↓2.25% (EG), ↔ (CG)
Hamilton, Paton & Hopkins (2006)	Competitive distance runners	N: 20 (EG = 10, CG = 10); Sex: M; Age: 28 ± 8 yrs (EG), 31 ± 6 yrs (CG); TE: 6.2 ± 4.7 yrs (EG), 8.5 ± 8.4 yrs (CG); ht: 178 ± 6 cm (EG), 178 ± 11 cm (CG); WT: 72 ± 11 kg (EG), 73 ± 10 kg (CG); VO_2_max: 66 ± 7 (EG), 66 ± 3 (CG) mL/kg/min	Length: 5–7 weeks; Freq: 1–3 times/week; 30 min per time	EG: HIRT + Regular Running Training CG: Regular Running Training	800 m, 1,500 m, 5 km Time-Trial Speed	800 m: ↑4.4% (EG), ↑0.8% (CG); 1,500 m: ↑4.1% (EG), ↑0.4% (CG); 5 km Speed: ↑2.2% (EG), ↑1.0% (CG)
Silva et al. (2022)	Professional soccer players	N: 18 (EG = 9, CG = 9); Sex: M; Age: 20.3 ± 0.6 yrs (EG), 20.4 ± 0.7 yrs (CG); TE: 6.0 ± 2.5 (EG) yrs, 5.3 ± 2.1 yrs (CG); ht: 173.0 ± 6.1 cm (EG), 175 ± 6.1 cm (CG); WT: 69.7 ± 6.5 kg (EG), 68.6 ± 6.5 kg (CG); BMI: 23.2 ± 1.4 (EG), 22.4 ± 1.7 (CG)	Length: 8 weeks; Freq: 3 HIRT + 2; technical/tactical times/week (EG), 5 technical/tactical times/week (CG)	EG: HIRT + Technique/Tactical Soccer Training CG: Technique/Tactical Soccer Training	YYIRT1, ST (10 m/20 m/30 m), RAST, COD	YYIRT1: ↑142 m (EG), ↔ (CG); ST (10 m): ↓0.06 s (EG), ↔ (CG); ST (20 m): ↓0.10 s (EG), ↔ (CG); ST (30 m): ↓0.24 s (EG), ↔ (CG); RAST: ↓0.12 s (EG), ↔ (CG); COD: ↓0.37 s (EG), ↔ (CG)
Hoff, Gran & Helgerud (2002)	National cross-country skiers	N: 19 (EG = 9, CG = 10); Sex: M; Age: 20.4 ± 4.3 yrs (EG), 19.2 ± 3.9 yrs (CG); TE: ≥5 yrs, ht: 175.7 ± 3.1 cm (EG), 183.0 ± 4.1 cm (CG); WT: 70.7 ± 4.5 kg (EG), 75.6 ± 5.4 kg (CG); VO_2_max: 69.7 ± 2.3 (EG), 69.1 ± 2.3 (CG) mL/kg/min	Length: 9 weeks; Freq: 3 times/week; 15 min per time	EG: HIRT + Endurance training CG: Endurance Training	1RM (Half-Squat), TTE, Exercise Economy, PF	1RM: ↑9.9% (EG), ↔ (CG); TTE: ↑56% (EG), ↑25% (CG); Exercise Economy: ↑(EG), ↔ (CG); PF: ↑(EG), ↔ (CG)
Hoff, Helgerud & Wisloeff (1999)	Elite cross-country skiers	N: 15 (EG = 8, CG = 7); Sex: F, Age: 17.8 ± 0.4 yrs (EG), 18.0 ± 0.4 yrs (CG); TE: NR; ht: 164.9 ± 1.9 cm (EG), 168.7 ± 1.4 cm (CG); WT: 56.8 ± 2.3 kg (EG), 63.9 ± 2.6 kg (CG); VO_2_max: 55.3 ± 2.2 (EG), 55.3 ± 1.6 (CG) mL/kg/min	Length: 9 weeks; Freq: 3 times/week; 8.5 ± 0.8 h per week (EG), 9.2 ± 1.2 h per week (CG)	EG: HIRT + Endurance training CG: Low-Intensity/General Strength Training + Endurance Training	1RM (Upper Limb), TTE, Skiing Economy, TPF	1RM: ↑14.5% (EG), ↔ (CG); TTE: ↑136% (EG), ↑57% (CG); Skiing Economy: ↑ (EG), ↔ (CG); TPF: ↓27% (EG), ↔ (CG)
Aagaard et al. (2011)	National cyclists	N: 14 (EG = 7, CG = 7); Sex: M; Age: 19.5 ± 0.8 yrs; TE: NR; ht: 180.7 ± 5.4 cm; WT: 70.7 ± 5.8 kg; VO_2_max: 73.5 ± 8.2 (EG), 71.5 ± 6.0 (CG) mL/kg/min	Length: 16 weeks; Freq: 2-3 times/week	EG: HIRT + Endurance Training CG: Endurance Training	MVC, RFD, 5min & 45 min Time-Trial Test, Cycling Economy	MVC: ↑12% (EG), ↔(CG); RFD: ↑20% (EG), ↔(CG); 5 min: ↑3–4% (all); 45 min: ↑8% (EG), ↔(CG); Cycling Economy: ↑ (CG), ↔ (EG)
Millet et al. (2002)	National and international-level triathletes	N: 15 (EG = 7, CG = 8); Sex: NR; Age: 24.3 ± 5.2 yrs (EG), 21.4 ± 2.1 yrs (CG); TE: 7.0 ± 2.6 yrs (EG), 6.6 ± 1.7 yrs (CG); ht: 175.4 ± 9.1 cm (EG), 175.4 ± 5.4 cm (CG); WT: 67.4 ± 8.8 kg (EG), 65.0 ± 7.4 kg (CG); VO_2_max: 69.7 ± 3.6 (EG), 67.6 ± 6.4 (CG) mL/kg/min	Length: 14 weeks; Freq: 2 times/week	EG: HIRT + Endurance Training CG: Endurance Training	1RM (Half-Squat, Calf Raise), Running Economy, Max Hopping Power	1RM ↑25% (EG), ↔ (CG); Running economy ↑ (EG), ↔ (CG); Max Hopping Power: ↔ (EG), ↓ (CG)
Sunde et al. (2010)	Professional cyclists	N: 13; Sex: 10M, 3F (EG:7M,1F, CG: 3M, 2F); Age: 29.9 ± 7.2 yrs (EG), 35.8 ± 11.8 yrs (CG); TE: NR; ht: 178 ± 8 cm (EG), 178 ± 13 (CG); WT: 72.5 ± 7.3 kg (EG), 75.4 ± 11.2 kg (CG); VO_2_max: 63.4 ± 6.0 (EG), 58.7 ± 8.8 (CG) mL/kg/min	Length: 8 weeks; Freq: 3 times/week	EG: HIRT + Cycling Training CG: Cycling Training	1RM (Half-Squat), RFD, Cycling Economy, Work Efficiency, TTE	1RM: ↑14.2% (EG), ↔ (CG); RFD: ↑16.7% (EG), ↔ (CG); Cycling Economy: ↑4.8% (EG), ↔ (CG); Work Efficiency: ↑4.8% (EG) >↑ (CG); TTE: ↑17.2% (EG), ↔ (CG)
Veliz et al. (2015)	National water polo players	*N* = 21(EG = 11, CG = 10); Sex: F; Age: 26.4 ± 4.3 yrs; TE: 10.6 ± 4.1 yrs; ht: 172.3 ± 5.8 cm; WT: 72.4 ± 8.5 kg; BF:19.5 ± 3.2%	Length:16 weeks; Freq: 2 times/week; concurrent in-water training: 5 sessions/week; 30 min per time	EG: HIRT + Water Polo Training CG: Water Polo Training	1RM (Full Squat), CMJ, VJ (In-Water Boost), PPO, TV, ST (20m Swim)	1RM: ↑20.99% (EG), ↔ (CG); CMJ: ↑8.66%, (EG), ↔ (CG); VJ: ↑12.02% (EG), ↔ (CG); PPO: ↑5.22% (EG), ↔ (CG); TV: ↑6.86% (EG), ↔ (CG); ST: ↓ 1.31% (EG), ↔ (CG)
Losnegard et al. (2011)	Elite cross-country skiers	*N* = 19; Sex: 11M, 8F (EG:6M,3F; CG:5M,5F); Age: 21.2 ± 3.2 yrs (EG), 21.7 ± 2.5 yrs (CG); TE: NR; ht: 176.7 ± 8.9 cm (EG), 173.3 ± 7.0 (CG); WT: 71.4 ± 10.2 kg (EG), 67.8 ± 10.6 kg (CG); VO_2_max (running): 64.7 ± 4.9 (EG), 64.6 ± 7.1 (CG) mL/kg/min; VO_2_max (skiing): 61.6 ± 5.5 (EG), 62.0 ± 9.2 (CG) mL/kg/min	Length: 12 weeks; Freq: 1–2 times/week	EG: HIRT + Endurance Training CG: Endurance Training	1RM (Half-Squat, Seated Pull-Down), CMJ, DP Power (5-min Ergometer), Time-Trial (Skate and DP), 100 m ST (Rollerski)	1RM (Pull-Down):↑19% (EG), ↔ (CG); 1RM (Half-Squat):↑12% (EG), ↔ (CG); CMJ: ↔ (EG), ↓6.2% (CG); DP Power:↑(EG), ↔ (CG); Time-Trial: ↑(EG), ↑ (CG); 100 m ST: ↓1.3% (EG), ↔ (CG)
Østerås, Helgerud & Hoff (2002)	Elite cross country skiers	*N* = 19 (EG = 10, CG = 9); Sex: M; Age: 21.0 ± 1.6 yrs (EG), 24.4 ± 5.0 yrs (CG); TE: ≥5 yrs; ht: 182.0 ± 2.8 cm (EG), 180.0 ± 3.8 (CG); WT: 77.8 ± 4.9 kg (EG), 74.0 ± 3.5 kg (CG); VO_2_peak: 63.1 ± 5.8 (EG), 59.2 ± 2.8 (CG) mL/kg/min	Length: 9 weeks; Freq: 3 times/week; 15 min per time	EG: HIRT + Endurance Training CG: Traditional Strength-Endurance Training + Endurance Training	1RM (Seated Pull-Down), TTE (Ski Ergometer), DP Economy, PPO	1RM: ↑20.9% (EG), ↔ (CG); TTE: ↑61% (EG), ↑6.4% (CG); DP Economy: ↑ (EG), ↔ (CG); PPO: ↑ (EG), ↔ (CG)
Vikmoen et al. (2017)	Well-trained female duathletes	N: 19 (EG = 11, CG = 8); Sex: F; Age: 30.3 ± 4.3 yrs; TE: NR; ht: 168 ± 5 cm; WT: 61.3 ± 4.8 kg; VO_2_max (Cycling): 54 ± 3 ml/kg/min; VO_2_max (Running): 53 ± 3 ml/kg/min	Length:11 weeks; Freq: 2 times/week	EG: HIRT + Endurance Training CG: Endurance Training	1RM (Half Squat); 5-min All-Out Cycling and Running Tests	1RM:↑45% (EG), ↔ (CG); 5-min Cycling:↑7%, ↔ (CG); 5-min Running:↑4.7% (EG), ↔ (CG)

**Notes.**

M Male F Female TE Training Experience ht height WT weight BF Body Fat Freq Frequency EG Experimental Group CG Control Group NR Not Reported HIRT High-Intensity Resistance Training 1RM 1 Repetition Maximum (maximum strength performance) TV Throwing Velocity VJ Vertical Jump height ST Sprint Time VO_2_max Maximal Oxygen Uptake BMI Body Mass Index (kg/m^2^) YYIRT1 Yo-Yo Intermittent Recovery Test—level 1 RAST Running-Based Anaerobic Sprint Test COD Change-Of-Direction TTE Time To Exhaustion PF Peak Force (N) TPF Time To Peak Force MVC Maximum Voluntary Contraction RFD Rate of Force Development CMJ Countermovement Jump PPO Peak Power Output (W) DP Double Poling VO_2_peak Peak Oxygen Uptake

Symbols (↑, ↓) indicate statistically significant changes between pre- and post-intervention (*p* < 0.05 or better); ↔ indicates no significant change.

**Table 3:** Quantitative summary of HIRT effects on athletic performance.

Outcome domain	n (Studies)	Typical change	Overall direction of findings	Representative findings
Maximal Strength (1RM, MVC)	10	↑ approximately 10–45%	Consistently Favors HIRT	1RM (bench press) increased by 10.53% and 1RM (full squat) by 14.21% in water polo players (Ramos Veliz et al., 2014); 1RM increased by 20.99% in national-level female water polo players (Veliz et al., 2015); seated pull-down 1RM increased by 20.9% in elite cross-country skiers (Østerås, Helgerud & Hoff, 2002); and half-squat 1RM increased by 45% in well-trained female duathletes (Vikmoen et al., 2017).
Power (CMJ, PPO, VJ, RFD, PF, TPF, DP)	9	↑approximately 5–20%; selected force-time measures also improved	Generally Favors HIRT	CMJ increased by 8.66%, VJ by 12.02%, and PPO by 5.22% following HIRT in water polo players (Veliz et al., 2015); RFD increased by 20% in cyclists (Aagaard et al., 2011) and by 16.7% in professional cyclists (Sunde et al., 2010); and time to peak force improved by 27% in elite female cross-country skiers (Hoff, Helgerud & Wisloeff, 1999).
Sport-Specific Velocity (TV)	2	↑ 2–7%	Favors HIRT	Throwing velocity increased by 2.76% in elite male water polo players (Ramos Veliz et al., 2014) and by 6.86% in national-level female water polo players (Veliz et al., 2015).
Sprint/Speed (ST)	4	↓approximately 1–2.5% or 0.06–0.24 s	Generally Favors HIRT	Sprint-related performance improved across several sports, including a 2.25% reduction in 20-m swim sprint time (Ramos Veliz et al., 2014), a 0.06–0.24 s reduction in 10–30 m sprint times (Silva et al., 2022), and a 1.3% improvement in 100-m rollerski sprint performance (Losnegard et al., 2011).
Change-of-Direction (COD) Speed	1	↓ 0.37s	Favors HIRT	Only one study assessed COD performance and reported a 0.37 s reduction following HIRT (Silva et al., 2022).
Endurance/Economy/ Time-Trial Performance	10	TTE: ↑ 17–136%; economy and time-trial outcomes generally improved or were maintained	Mostly Favors HIRT	TTE increased by 136% in elite cross-country skiers (Hoff, Helgerud & Wisloeff, 1999), by 61% in elite skiers using ski ergometer testing (Østerås, Helgerud & Hoff, 2002), and by 17.2% in professional cyclists (Sunde et al., 2010); YYIRT1 performance improved by 142 m in professional soccer players (Silva et al., 2022); lactate-threshold speed increased by 3.5% in competitive distance runners (Hamilton, Paton & Hopkins, 2006); and 5-min all-out cycling and running performance improved in well-trained female duathletes (Vikmoen et al., 2017).

**Notes.**

CMJ countermovement jump PPO peak power output VJ vertical jump ST sprint test TV throwing velocity COD change of direction TTE time-to-exhaustion 1RM one-repetition maximum MVC maximal voluntary contraction CMJ countermovement jump PPO peak power output VJ vertical jump RFD rate of force development PF peak force TPF time to peak force DP double poling power RAST Running-Based Anaerobic Sprint Test YYIRT1 Yo-Yo Intermittent Recovery Test Level 1 ↔ no significant change ↑ increase

Values represent ranges reported across studies; effect sizes were included where available. Each study was counted once within an outcome domain if it assessed at least one relevant outcome in that domain; a study contributing outcomes to more than one domain was counted once in each applicable domain. Direction entries reflect the overall direction of evidence, based primarily on statistically significant within-group pre–post changes, and do not, by themselves, demonstrate statistically significant between-group differences.

## Results

### Study selection

The study selection process is illustrated in Fig. 1. The initial search yielded a total of 1,543 records, comprising 403 references identified from four primary electronic databases (PubMed, Scopus, Web of Science, and SPORTDiscus) and 1,140 records identified from other sources (primarily citation tracking *via* Google Scholar). Following the removal of 364 duplicates using Zotero, 1,179 titles and abstracts were screened for relevance. Of these, 1,049 records were excluded for failing to meet the inclusion criteria regarding population, intervention type, or study design. Consequently, 130 full-text articles were retrieved and assessed for eligibility. Ultimately, 12 studies satisfied all inclusion requirements and were included in the final synthesis. The study selection process adhered to the PRISMA 2020 guidelines (Page et al., 2021). Reasons for exclusion at the full-text stage were documented, with the most frequent grounds being: recruitment of non-elite populations, intervention intensities below the HIRT threshold (<75% 1RM), intervention durations shorter than 4 weeks, or insufficient methodological quality (PEDro score ≤ 4).

**Figure 1:**
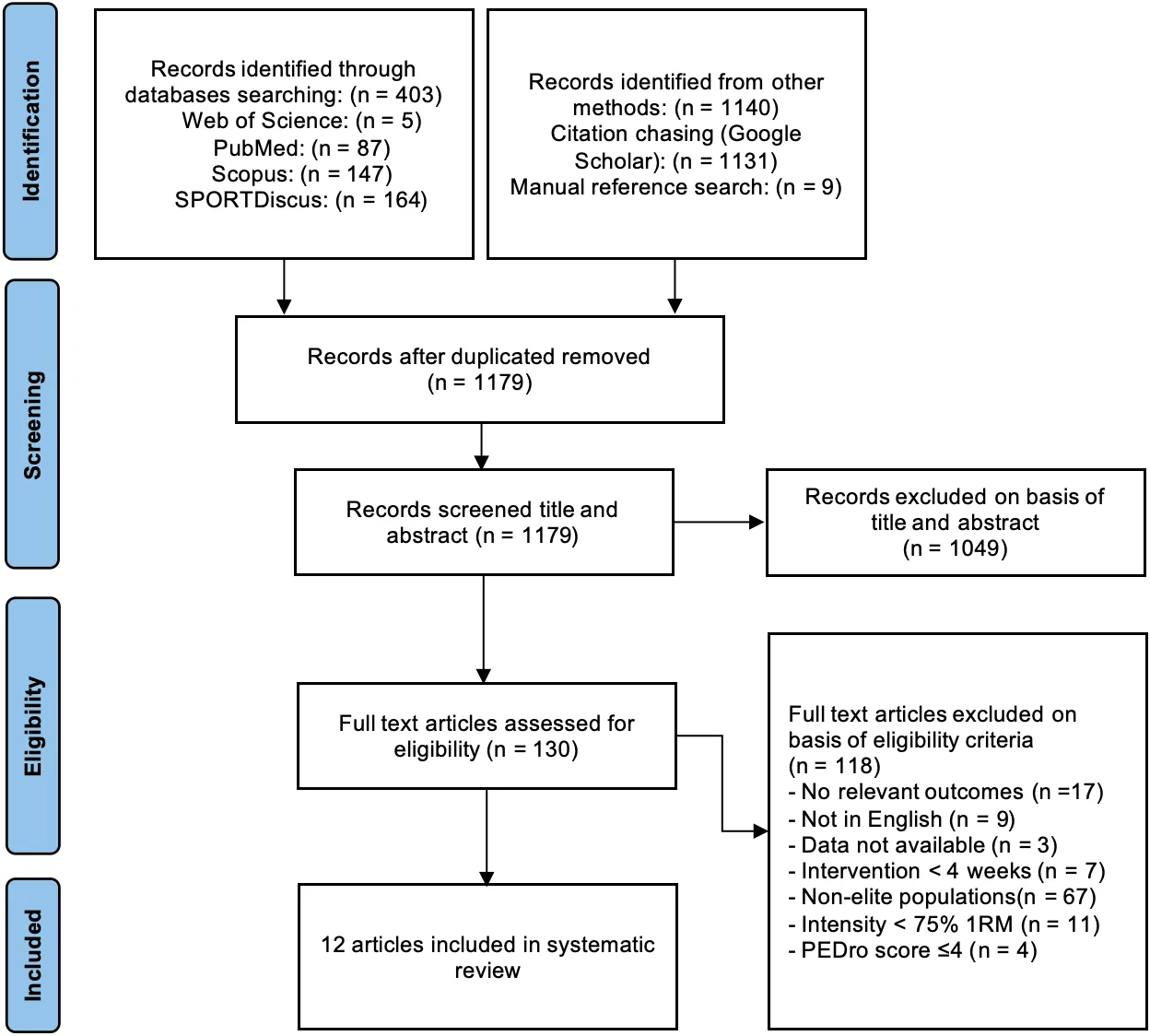
PRISMA flow chart of the study selection process. PRISMA 2020 flow diagram of the study selection process. The diagram illustrates the database search strategy, screening of records, eligibility assessment, and final inclusion of studies for the systematic review.

### Study quality assessment

The methodological quality of the included studies was evaluated using the PEDro scale. All 12 studies demonstrated good methodological quality, with scores ranging from 5 to 6 out of 10. Specifically, two studies (Aagaard et al., 2011; Silva et al., 2022), achieved a score of 6, while the remaining ten studies (Hoff, Helgerud & Wisloeff, 1999; Hoff, Gran & Helgerud, 2002; Millet et al., 2002; Østerås, Helgerud & Hoff, 2002; Hamilton, Paton & Hopkins, 2006; Sunde et al., 2010; Losnegard et al., 2011; Ramos Veliz et al., 2014; Veliz et al., 2015; Vikmoen et al., 2017) received a score of 5. The scores were primarily limited by the challenge of double blinding in resistance training interventions (*i.e.,* participants and therapists are aware of the training modality), which typically precludes achieving maximum PEDro scores. By strictly excluding studies with moderate or low quality (scores ≤ 4), this review ensures that the synthesized findings are derived from trials with established internal validity.

### Participant characteristics

A cumulative total of 219 athletes were included across the 12 reviewed studies, with sample sizes ranging from 13 (Sunde et al., 2010) to 27 (Ramos Veliz et al., 2014) (mean: 18.3; median: 19). The recruited populations represented a diverse array of sporting disciplines, primarily endurance and team sports, including cross-country skiing and cycling (Hoff, Helgerud & Wisloeff, 1999; Hoff, Gran & Helgerud, 2002; Østerås, Helgerud & Hoff, 2002; Sunde et al., 2010; Aagaard et al., 2011; Losnegard et al., 2011), multi-endurance disciplines such as running, triathlon, and duathlon (Millet et al., 2002; Hamilton, Paton & Hopkins, 2006; Vikmoen et al., 2017), soccer (Silva et al., 2022), and water polo (Ramos Veliz et al., 2014; Veliz et al., 2015). The study population comprised six studies exclusively recruiting male athletes (Hoff, Gran & Helgerud, 2002; Østerås, Helgerud & Hoff, 2002; Hamilton, Paton & Hopkins, 2006; Aagaard et al., 2011; Ramos Veliz et al., 2014; Silva et al., 2022), three focusing solely on female athletes (Hoff, Helgerud & Wisloeff, 1999; Veliz et al., 2015; Vikmoen et al., 2017), and two including both male and female athletes (Sunde et al., 2010; Losnegard et al., 2011); in one study (Millet et al., 2002), participant sex was not clearly reported. These data indicate a male predominance in the available HIRT literature, although female-only and mixed-sex cohorts were also represented. Participants’ ages ranged from 17.8 (Hoff, Helgerud & Wisloeff, 1999) to 35.8 years (Sunde et al., 2010), with the majority falling between 19 and 30 years, corresponding to the prime physiological window for competitive athletic performance (Aagaard et al., 2011; Vikmoen et al., 2017).

Regarding anthropometric characteristics, reported body mass ranged from 56.8 ± 2.3 kg (Hoff, Helgerud & Wisloeff, 1999) to 81.4 ± 8.4 kg (Ramos Veliz et al., 2014). While Body Mass Index (BMI) was explicitly reported in only one study (Silva et al., 2022), estimates derived from height and weight data indicated values between 20.9 and 24.5 kg/m^2^, consistent with athletic body compositions rather than sedentary norms. Body fat percentage was reported in two studies, showing values of 12.4% and 19.5%, further reflecting the trained status of the cohorts.

All participants met the criteria for elite or highly trained status (Jeukendrup, Craig & Hawley, 2000). Several studies explicitly reported training histories of at least five years (Hoff, Gran & Helgerud, 2002; Østerås, Helgerud & Hoff, 2002), and some cohorts exceeded ten years of sport-specific training experience (Ramos Veliz et al., 2014; Veliz et al., 2015). In studies where training experience was not reported, elite or highly trained status was supported by competitive level and physiological profile. This classification is physiologically substantiated by the reported aerobic capacity values: VO_2_max (or VO_2_peak) ranged from approximately 54 mL/kg/min in duathletes (Vikmoen et al., 2017) to 73.5 mL/kg/min in cyclists (Aagaard et al., 2011), indicating superior conditioning levels characteristic of national and professional-level competitors.

### Intervention characteristics

Intervention durations varied considerably across the 12 studies, ranging from 5 weeks (Hamilton, Paton & Hopkins, 2006) to 18 weeks (Ramos Veliz et al., 2014), with a mean duration of approximately 11.3 weeks. This timeframe aligns with standard mesocycles designed to elicit neuromuscular adaptations without inducing overtraining in competitive populations. Training frequency ranged from 1 to 3 sessions per week across the included studies. Five studies prescribed three weekly sessions (Hoff, Helgerud & Wisloeff, 1999; Hoff, Gran & Helgerud, 2002; Østerås, Helgerud & Hoff, 2002; Sunde et al., 2010; Silva et al., 2022), whereas several others used a bi-weekly format (Aagaard et al., 2011; Ramos Veliz et al., 2014; Veliz et al., 2015; Vikmoen et al., 2017). Hamilton, Paton & Hopkins (2006) reported a variable frequency of 1–3 sessions per week. Session duration, where explicitly reported, was generally brief to moderate rather than prolonged, ranging from approximately 15 to 30 min per session in several studies (Hoff, Gran & Helgerud, 2002; Østerås, Helgerud & Hoff, 2002; Hamilton, Paton & Hopkins, 2006; Ramos Veliz et al., 2014; Veliz et al., 2015).

Regarding training protocols, all interventions were judged to meet the operational definition of HIRT (intensities ≥75% 1RM). A subset of studies implemented Maximal Strength Training protocols, characterized by near-maximal loads (≥85% 1RM) performed for fewer repetitions (*e.g.*, 3–5 sets of 3–6 reps) to prioritize neural adaptations (Østerås, Helgerud & Hoff, 2002; Sunde et al., 2010; Vikmoen et al., 2017). Exercise selection primarily focused on multi-joint, compound movements such as squats, bench presses, and deadlifts, supplemented by sport-specific actions like double-poling or overhead throwing. Across all included studies, HIRT was not implemented as an isolated intervention; rather, it was incorporated into athletes’ existing sport-specific training regimens. Accordingly, the findings of the present review reflect the effects of HIRT within concurrent training programs rather than the isolated effect of HIRT alone. Periodization strategies varied, with some studies employing undulating models (Losnegard et al., 2011) and others following linear progressive overload tailored to the athletes’ competitive calendars.

### Outcome characteristics

#### Effects of HIRT training on muscular strength

Muscular strength outcomes were reported in ten studies, assessed primarily through 1RM or maximum voluntary contraction (MVC) (Hoff, Helgerud & Wisloeff, 1999; Hoff, Gran & Helgerud, 2002; Millet et al., 2002; Østerås, Helgerud & Hoff, 2002; Sunde et al., 2010; Aagaard et al., 2011; Losnegard et al., 2011; Ramos Veliz et al., 2014; Veliz et al., 2015; Vikmoen et al., 2017).

Among these, 1RM strength significantly increased in multiple movements, with percentage improvements ranging from approximately 10% to 45% across interventions lasting 8 to 18 weeks in most cases (Hoff, Gran & Helgerud, 2002; Sunde et al., 2010; Ramos Veliz et al., 2014; Vikmoen et al., 2017). Specifically, Ramos Veliz et al. (2014) reported a 10.5% increase in bench press and a 14.2% increase in full squat 1RM in elite water polo players, whereas the control group showed no significant changes. Even greater gains were observed by Vikmoen et al. (2017), who documented a 45% increase in 1RM half-squat performance in female duathletes after 11 weeks of concurrent training.

Similarly, Østerås, Helgerud & Hoff (2002) observed a 20.9% increase in 1RM pull-down strength in cross-country skiers. Losnegard et al. (2011) found a 19% increase in 1RM seated pull-down strength after combined endurance and HIRT. While Aagaard et al. (2011) noted improvements in MVC in trained cyclists, the study did not report 1RM as an outcome measure.

Additional strength gains were also reported in elite female skiers, triathletes, and female water polo players, including a 14.5% increase in upper limb 1RM (Hoff, Helgerud & Wisloeff, 1999), a 25% increase in half-squat strength (Millet et al., 2002), and a 20.99% increase in full squat 1RM (Veliz et al., 2015). Despite differences in sport type and testing modality, the collective findings indicate that HIRT effectively enhances maximal muscular strength, primarily through neural adaptations, without clear evidence of compromising endurance-related performance in elite populations.

#### Effects of HIRT on power

Power-related outcomes were evaluated in nine studies (Hoff, Helgerud & Wisloeff, 1999; Hoff, Gran & Helgerud, 2002; Millet et al., 2002; Østerås, Helgerud & Hoff, 2002; Sunde et al., 2010; Aagaard et al., 2011; Losnegard et al., 2011; Ramos Veliz et al., 2014; Veliz et al., 2015), utilizing assessment methods such as countermovement jump (CMJ), peak power output (PPO), and rate of force development (RFD), as well as related force-time and sport-specific power measures such as peak force (PF), time to peak force (TPF), vertical jump (VJ), and double-poling (DP) power. While some of these outcomes reflect explosive performance indirectly rather than mechanical power in the strict biomechanical sense, they are commonly used in sport performance research to represent neuromuscular power-related adaptations.

Despite this methodological distinction, significant improvements in power production were consistently reported. For instance, Ramos Veliz et al. (2014) observed a 6.9% increase in CMJ height in elite water polo players following 18 weeks of HIRT, whereas the control group showed no significant changes. Similarly, Veliz et al. (2015) reported an 8.66% increase in CMJ, a 12.02% increase in in-water vertical jump (VJ), and a 5.22% increase in peak power output (PPO) in national-level female water polo players.

In endurance-oriented sports, Aagaard et al. (2011) reported a 20% increase in RFD in trained cyclists, while Sunde et al. (2010) observed a 16.7% increase in RFD in professional cyclists. Hoff, Gran & Helgerud (2002) reported improved peak force, and Hoff, Helgerud & Wisloeff (1999) documented a 27% improvement in time to peak force in elite female cross-country skiers. Østerås, Helgerud & Hoff (2002) found that upper-body HIRT increased PPO in cross-country skiers, whereas Losnegard et al. (2011) reported improved double-poling power after combined endurance and HIRT. Millet et al. (2002) also assessed maximal hopping power, although this outcome remained unchanged in the intervention group.

None of the included studies reported negative changes in power-related outcomes. These findings suggest that high-intensity loading (≥85% 1RM), traditionally associated with maximal strength, effectively enhances neuromuscular power through improved motor unit discharge rates and intra-muscular coordination.

#### Effects of HIRT on sport-specific throwing velocity

Sport-specific throwing velocity was assessed in two studies involving elite water polo players (Ramos Veliz et al., 2014; Veliz et al., 2015). Significant improvements were reported in both cases. In male water polo players, throwing velocity increased by 2.76% following 18 weeks of HIRT, whereas the control group showed no significant change (Ramos Veliz et al., 2014). In female water polo players, a 6.86% increase in throwing velocity was observed after 16 weeks of HIRT, with no corresponding improvement in the control group (Veliz et al., 2015).

These findings suggest that HIRT may positively influence sport-specific throwing velocity in aquatic team-sport settings. Nevertheless, because the available evidence is limited to two water polo studies, broader generalization to other sports requiring throwing or striking actions should be made cautiously.

#### Effects of HIRT on speed

Speed-related performance was evaluated in four studies using land-based linear sprints (10–30 m), the Running-based Anaerobic Sprint Test (RAST) (Silva et al., 2022), rollerski sprints (Losnegard et al., 2011), or sport-specific aquatic trials (Ramos Veliz et al., 2014; Veliz et al., 2015).

In land-based assessments, Silva et al. (2022) observed consistent improvements in sprint performance following an 8-week HIRT program. Specifically, elite soccer players achieved a reduction of 0.24 s in 30-m sprint time and a 0.12 s reduction in RAST time.

In aquatic environments, HIRT demonstrated effective transfer to sport-specific speed. Ramos Veliz et al. (2014) reported a significant 2.25% reduction in 20-m swim sprint time in elite water polo players, whereas the control group (in-water training only) showed no performance changes. Similarly, Veliz et al. (2015) documented a 1.31% improvement in swim sprint performance under similar loading protocols.

Conversely, results in endurance-dominant sports were more limited. Losnegard et al. (2011) reported a 1.3% improvement in 100-m rollerski sprint performance after 12 weeks of concurrent training, whereas the control group showed no clear change. Hamilton, Paton & Hopkins (2006) were not included in this speed-specific domain because their outcomes primarily reflected endurance and time-trial performance rather than maximal sprint speed.

Collectively, while the magnitude of improvement in sprint performance (approx. 1–2.5% or 0.06–0.24 s) is generally smaller than gains seen in maximal strength (approximately 10–45%), these marginal gains are critically important in elite competitive contexts where performance may be determined by fractions of a second.

#### Effects of HIRT on preplanned change-of-direction speed

Outcomes related to preplanned change-of-direction (COD) speed were reported in only one study. Silva et al. (2022) assessed soccer-specific COD performance using a timed field-based shuttle run test. Because this assessment was conducted under a closed, preplanned condition, it should be interpreted as a measure of COD speed rather than true agility, which additionally involves perceptual-cognitive processing in response to unpredictable stimuli. Following the 8-week HIRT intervention, the experimental group achieved a significant 0.37-second reduction in completion time, whereas the control group showed no change from baseline.

Although evidence is currently limited to this single trial, the finding suggests that HIRT may improve preplanned COD speed in field-sport athletes. This effect may be explained by improvements in eccentric strength, which enhance deceleration capacity, and concentric power, which support rapid re-acceleration during directional changes. However, the present review does not provide evidence regarding the effect of HIRT on true perceptual-cognitive agility.

#### Effects of HIRT on endurance performance and efficiency

Endurance performance and exercise economy were assessed in ten studies using sport-specific protocols. Contrary to concerns that hypertrophy might impair endurance, HIRT was generally associated with improvements in performance markers without clear detrimental effects on VO_2_max.

In cross-country skiing, Østerås, Helgerud & Hoff (2002) reported a 61% increase in Time to Exhaustion (TTE) on a ski ergometer following 9 weeks of upper-body maximal strength training, alongside significant gains in peak power output. Similarly, Hoff, Gran & Helgerud (2002) observed improved skiing work economy (reduced oxygen cost at submaximal workloads) despite no change in VO_2_max, highlighting the role of mechanical efficiency. Hoff, Helgerud & Wisloeff (1999) also reported a 136% increase in TTE in elite female cross-country skiers; however, as discussed above, such exceptionally large changes should be interpreted cautiously because open-ended exhaustion tests are particularly sensitive to baseline values, learning effects, motivational influences, and stopping criteria.

In concurrent training for duathletes, Vikmoen et al. (2017) documented robust improvements in all-out performance tests: the HIRT group achieved a 7.0% improvement in 5-min cycling power and a 4.7% improvement in 5-min running performance, whereas the endurance-only control group showed no significant changes. In cyclists, Sunde et al. (2010) reported improvements in cycling economy, work efficiency, and TTE, while Aagaard et al. (2011) found improved 45-min time-trial performance despite no clear change in VO_2_max. Losnegard et al. (2011) also reported improved double-poling time-trial performance after combined endurance and HIRT.

Improvements were also evident in team sports and distance running. Silva et al. (2022) showed a significant 142-meter increase (approx. +9%) in the Yo-Yo Intermittent Recovery Test Level 1 (YYIRT1) among professional soccer players. Millet et al. (2002) observed significant enhancements in submaximal running economy in triathletes, and Hamilton, Paton & Hopkins (2006) noted improved performance in 800 m, 1,500 m, and 5 km time trials.

Collectively, these findings indicate that HIRT enhances endurance performance primarily by improving movement economy (lower energy cost for the same power output) and neuromuscular efficiency (delayed recruitment of Type II fibers), rather than by altering cardiovascular capacity (VO_2_max) (Hoff, Helgerud & Wisloeff, 1999; Aagaard et al., 2011). However, endurance-related outcomes should be interpreted more cautiously than strength outcomes, particularly when exceptionally large TTE changes are used to characterize adaptation.

### Quantitative summary of findings

To provide a comprehensive overview of the magnitude of HIRT-induced adaptations, a quantitative synthesis of the included studies is presented in Table 3. This summary aggregates the typical percentage changes, the overall direction of findings, and representative findings across key outcome domains. The synthesized data highlight a consistent trend: HIRT elicits substantial improvements in maximal strength (approximately 10–45%) and power (approximately 5–20%), with no clear evidence of detrimental effects on endurance-related outcomes. Furthermore, favorable adaptations are observed in sport-specific velocity and preplanned change-of-direction (COD) speed, reinforcing the functional transfer of high-intensity loading to competitive performance. However, the findings should be interpreted within the context of concurrent training designs and varying levels of certainty across outcome domains.

## Discussion

The central finding of this systematic review is that High-Intensity Resistance Training (HIRT, ≥75% 1RM) was associated with consistent and substantial improvements in maximal strength (ranging from 10% to 45%) and neuromuscular power (∼5–20%) in elite athletes, with no clear evidence of detrimental effects on endurance-related outcomes. Endurance-related variables, including movement economy, time-trial performance, and time to exhaustion (TTE), also generally favored HIRT, although the exceptionally large TTE gains reported in some studies should be interpreted cautiously given the methodological sensitivity of open-ended exhaustion tests.

Unlike previous reviews that have largely examined general resistance training in recreational or moderately trained populations, this review specifically examines the performance effects of HIRT in elite cohorts. This distinction is critical, as elite athletes typically face a relative ceiling for adaptation. In this respect, the present findings extend recent evidence suggesting that resistance training can enhance sport-specific performance in elite athletes (Makaruk et al., 2024), while also aligning with more contemporary concurrent-training literature indicating that strength-oriented stimuli do not inevitably impair endurance-related adaptation (Huiberts, Wüst & Van der Zwaard, 2024). Mechanistically, the observed pattern is consistent with current physiological models emphasizing neuromuscular adaptations such as improved motor unit recruitment, discharge behavior, and force transmission efficiency as key contributors to performance enhancement (Lecce et al., 2026).

An important methodological consideration is that none of the included studies examined HIRT as an isolated intervention. In all cases, HIRT was implemented alongside ongoing sport-specific training, meaning that the present review synthesizes evidence from concurrent training programs rather than from isolated HIRT interventions. Consequently, the observed improvements in strength, power, sprint, and endurance-related outcomes should be interpreted as the additive effects of HIRT within a broader training environment. Although this does not diminish the practical relevance of HIRT for elite athletes, it limits causal attribution, as the specific contribution of HIRT cannot be fully disentangled from the accompanying sport-specific training stimulus. Accordingly, the findings of this review should be interpreted as likely low-to-moderate certainty evidence for the performance contribution of HIRT within concurrent training settings, rather than as definitive proof of the isolated effect of HIRT alone.

### Effects of HIRT on muscular strength

HIRT has been consistently associated with substantial improvements in muscular strength across elite athletes, overcoming the relative ceiling for adaptation typically observed in highly trained populations. In the reviewed studies, high-load interventions led to significant improvements in both upper- and lower-body strength. Ramos Veliz et al. (2014), Sunde et al. (2010) and Vikmoen et al. (2017) reported substantial increases ranging from 10% to 45% in 1RM squat or bench press following concurrent HIRT interventions of varying duration.

The magnitude of these gains is practically meaningful. For instance, the 14.2% increase in lower-body strength reported by Ramos Veliz et al. (2014), in elite water polo players directly addresses sport-specific demands, such as the eggbeater kick. Furthermore, the transfer of upper-body strength to performance outcomes—such as throwing velocity—was explicitly confirmed, aligning with findings in other power-based sports like sprint kayaking (McKean & Burkett, 2014) and handball (Marques et al., 2007). These parallels suggest that HIRT effectively enhances the force-generating capacity required for high-velocity sporting actions.

Mechanistically, these strength gains are primarily attributed to neural adaptations rather than structural morphological changes. As highlighted by Aagaard et al. (2011), Hoff, Helgerud & Wisloeff (1999), HIRT protocols utilizing loads of ≥75% 1RM with few repetitions maximize neural drive, including enhanced motor unit recruitment, synchronization, and firing frequency (rate coding). Recent physiological reviews likewise support the view that neural adaptations—including enhanced recruitment behavior, discharge properties, and force transmission efficiency—are central to early and functionally relevant strength gains following resistance training (Lecce et al., 2026). Although HIRT may also induce selective hypertrophy of Type II (fast-twitch) fibers (Aagaard et al., 2002), several included studies (Østerås, Helgerud & Hoff, 2002) observed strength gains without significant changes in total body mass. This indicates that HIRT improves ‘relative strength’—a critical attribute for elite athletes who must optimize power-to-weight ratios.

This distinction is vital for coaching practice. Recent reviews in elite and well-trained populations suggest that high-load resistance training remains an effective strategy for improving strength-related performance when the objective is to maximize neuromuscular function rather than hypertrophy alone (Makaruk et al., 2024; Currier et al., 2026). Accordingly, the strength improvements observed in the present review are most plausibly explained by optimization of neuromuscular output from existing muscle mass, although some contribution from structural adaptation cannot be excluded.

### Effects of HIRT on power

HIRT has been shown to significantly enhance neuromuscular power in elite athletes, particularly in tasks requiring rapid force production such as the countermovement jump (CMJ). Improvements in these domains were consistently reported in the reviewed studies, highlighting the transferability of gym-based strength to field performance. For instance, Veliz et al. (2015) observed an 8.7% increase in CMJ height following a 16-week resistance training program in elite female water polo players. Similarly, Ramos Veliz et al. (2014) reported comparable gains in male counterparts, with a 6.9% increase in vertical jump, despite the high baseline level of these athletes.

A dose–response relationship appears to exist between maximal strength gains and power outcomes. As noted in the broader literature, substantial increases in lower body 1RM are frequently correlated with improvements in acceleration and jump height (Silva, Nassis & Rebelo, 2015). In the context of this review, the concurrent improvements in strength (10–45%) and power (∼5–20%) suggest that increased maximal force availability serves as an important mechanical foundation for power production (P = F * V). This interpretation is also consistent with recent evidence indicating that resistance training can improve sport-specific performance in elite athletes when neuromuscular qualities are effectively targeted (Makaruk et al., 2024).

Mechanistically, these improvements are driven by neural factors rather than hypertrophy alone. As demonstrated by Aagaard et al. (2011), Sunde et al. (2010), and Hoff, Gran & Helgerud (2002), HIRT can improve rate of force development (RFD) and force-time characteristics relevant to explosive performance. The primary drivers include increased neural drive to high-threshold motor units, improved inter-muscular coordination, and enhanced motor unit synchronization (Aagaard et al., 2002; Aagaard et al., 2011). Recent physiological reviews likewise support the view that resistance training-induced improvements in explosive performance are strongly mediated by neural adaptations, including altered recruitment behavior and discharge properties, rather than hypertrophy alone (Lecce et al., 2026).

It is important to emphasize the role of ‘ballistic intent’. Although the actual movement velocity during HIRT (≥75% 1RM) is slow due to the heavy load, the athlete’s distinct intention to move the load as fast as possible recruits the same fast-twitch (Type II) motor units required for explosive sport actions. This aligns with Henneman’s size principle, whereby heavy loads are necessary to fully activate the high-threshold motor units responsible for power output. Thus, HIRT provides a unique stimulus that complements, rather than replaces, traditional high-velocity plyometric training.

### Effects of HIRT on sport-specific throwing velocity

The available evidence suggests that HIRT may improve sport-specific throwing velocity in elite aquatic athletes. Ramos Veliz et al. (2014) reported a 2.76% increase in throwing velocity in elite male water polo players after 18 weeks of HIRT, while Veliz et al. (2015) observed a 6.86% improvement in national-level female water polo players following a 16-week intervention. In both studies, the control groups showed no corresponding performance gains, indicating that HIRT may contribute meaningfully to explosive overhead sport actions.

Mechanistically, these improvements likely depend on more than isolated upper-limb strength. Throwing velocity in water polo requires effective force generation and transmission through the entire kinetic chain, including lower-limb support, trunk stiffness, and rapid upper-limb acceleration. Therefore, the observed improvements in throwing velocity are consistent with the broader pattern of HIRT-induced gains in maximal strength and neuromuscular power. Nevertheless, because the current evidence is limited to two water polo studies, the transferability of this effect to other throwing- or striking-based sports remains to be established.

### Effects of HIRT on speed

Although HIRT is often prioritized for maximal force development, our review confirms that these adaptations can transfer to sport-specific speed, albeit with a smaller magnitude of improvement (approximately 1–2.5%, or 0.06–0.24 s) compared to strength gains (>10%).

In aquatic sports, where drag forces are high, HIRT appears particularly effective. Ramos Veliz et al. (2014) reported a significant 2.25% reduction in 20-m swim sprint time in elite male water polo players following 18 weeks of HIRT. Veliz et al. (2015) observed similar trends in elite females (1.31% improvement), suggesting that HIRT effectively enhances propulsive force generation in water regardless of gender. In land-based field sports, Silva et al. (2022) demonstrated that an 8-week HIRT program reduced 30-m sprint times by 0.24 s in elite soccer players, significantly outperforming conventional field training. Conversely, in endurance-dominant sports like cross-country skiing, speed gains were present (*e.g.*, 1.3% improvement in 100-m sprint (Losnegard et al., 2011)) but were more modest, highlighting the challenge of transferring strength to high-velocity movements in highly trained aerobic athletes.

Mechanistically, the improvement in speed is likely driven by neural adaptations that enhance Rate of Force Development (RFD). As noted by Aagaard et al. (2011), increases in maximal contractile strength (MVC) allow athletes to generate greater ground reaction forces (GRF) within the short contact times of sprinting (<100 ms). Furthermore, HIRT increases the stiffness of the musculotendinous unit (Sunde et al., 2010), which improves the storage and recoil of elastic energy—a critical factor for stride efficiency.

However, it must be acknowledged that the magnitude of speed improvement is modest relative to the large gains in 1RM strength. This discrepancy can be explained by the principle of mechanical specificity. Traditional HIRT exercises (*e.g.*, squat, deadlift) primarily involve vertical force vectors, whereas sprinting requires substantial horizontal force application. While increased vertical force capacity contributes to acceleration (overcoming inertia), the direct transfer to maximum velocity phases is mechanically limited (Seitz et al., 2014). Therefore, for elite athletes, HIRT serves as a foundational stimulus that increases the potential for speed, which must then be realized through sport-specific plyometric or technical training.

### Effects of HIRT on Preplanned Change-of-Direction (COD) speed

While change-of-direction (COD) speed and agility are related, they are distinct constructs. COD speed refers to the physical ability to decelerate and re-accelerate through a preplanned movement pattern, whereas agility additionally incorporates perceptual-cognitive processing in response to an external stimulus (Sheppard & Young, 2006). In this review, the available evidence supports a possible positive transfer of HIRT to preplanned COD speed, particularly in field sports. Silva et al. (2022) reported a significant 0.37 s reduction in shuttle-run time in elite soccer players following 8 weeks of HIRT. Because this test was conducted under closed, preplanned conditions, it should be interpreted as a measure of COD speed rather than true agility. This improvement is practically meaningful in match scenarios where creating separation from an opponent relies on fractions of a second.

Although the number of studies assessing COD was limited to a single trial, the mechanisms of improvement are physiologically consistent. COD performance is heavily dependent on eccentric strength (for rapid deceleration/braking) and concentric power (for re-acceleration) (Spiteri et al., 2013). The substantial gains in lower-limb maximal strength (10–45%) observed in this review provide the force reserve necessary to absorb high ground reaction forces during the braking phase of a turn.

Furthermore, improvements in core stability—cited in aquatic studies like Veliz et al. (2015)—likely contribute to better postural control during rapid transitions. By enhancing the rigidity of the torso, HIRT ensures that force generated by the legs is more efficiently transmitted to movement velocity rather than being dissipated through trunk instability. Thus, while HIRT exercises (*e.g.*, squats) are often performed in the sagittal plane, the resulting neuromuscular adaptations may support the multi-planar demands of elite sport maneuvering. However, the present review does not provide evidence regarding the effect of HIRT on true perceptual-cognitive agility. This distinction is reinforced by recent work from Gandouzi et al. (2025), who developed the Team Agility Plus Test (TAPT) to address the ecological validity limitations of traditional horizontal-only, preplanned assessments in multidirectional sport contexts (Gandouzi et al., 2025).

### Effects of HIRT on endurance and efficiency

Although traditionally viewed as incompatible with endurance performance due to the hypothetical “interference effect”, the available evidence in this review suggests that HIRT elicits beneficial effects on both endurance capacity and movement economy in elite athletes. Several included studies reported substantial improvements in Time to Exhaustion (TTE) and time-trial performance following HIRT interventions. This interpretation is broadly consistent with more recent concurrent training literature, which indicates that the interaction between strength- and endurance-oriented stimuli may depend on training status, sex, and program design rather than reflecting a uniform negative interference effect (Huiberts, Wüst & Van der Zwaard, 2024).

Magnitude of improvements: Østerås, Helgerud & Hoff (2002) documented a 61% increase in TTE during double-poling ergometry in elite cross-country skiers following 9 weeks of upper-body HIRT. Similarly, Vikmoen et al. (2017) reported significant gains in female duathletes, with a 7.0% improvement in 5-min maximal cycling power and a 4.7% improvement in running performance. In intermittent sports, Silva et al. (2022) showed that HIRT improved Yo-Yo Intermittent Recovery Test (Level 1) performance by 142 m (9%) in elite soccer players, indicating enhanced fatigue resistance during repeated high-intensity efforts. However, the exceptionally large TTE gains reported in some studies should be interpreted cautiously. In particular, the 136% and 61% increases reported in elite cross-country skiers were substantially larger than the relative improvements observed in other outcome domains and may partly reflect the methodological sensitivity of open-ended exhaustion tests (Hoff, Helgerud & Wisloeff, 1999; Østerås, Helgerud & Hoff, 2002). Compared with fixed-distance or fixed-duration performance tests, TTE assessments are more susceptible to baseline effects, learning effects, motivational influences, and variation in stopping criteria.

Economy and efficiency: These performance gains appear to be driven by improvements in movement economy (reduced oxygen cost at a fixed velocity) rather than cardiovascular expansion. Hoff, Gran & Helgerud (2002) and Losnegard et al. (2011) demonstrated similar enhancements in skiing economy. In several reviewed studies (Hoff, Helgerud & Wisloeff, 1999; Hoff, Gran & Helgerud, 2002; Østerås, Helgerud & Hoff, 2002; Sunde et al., 2010; Aagaard et al., 2011; Vikmoen et al., 2017), VO_2_max remained unchanged, reinforcing the conclusion that performance improvements were mediated by neuromuscular rather than cardiorespiratory adaptations.

Underlying mechanisms: The primary mechanism explaining this ‘economy effect’ is the delayed recruitment of Type II muscle fibers. Increases in maximal strength (1RM) mean that each submaximal stride or stroke represents a lower percentage of the athlete’s maximal force capability. This allows the athlete to rely longer on fatigue-resistant Type I fibers before recruiting less efficient Type II fibers, thereby delaying metabolic acidosis (Paton & Hopkins, 2005; Aagaard et al., 2011). Additionally, HIRT increases the stiffness of the musculotendinous unit, allowing for more efficient storage and release of elastic energy during the stretch-shortening cycle (Hoff, Helgerud & Wisloeff, 1999).

Inconsistent findings & dosage: It is worth noting that not all research yields positive results. For instance, some external studies (Levin, Mcguigan & Laursen, 2009) reported no endurance gains in cyclists, potentially due to insufficient training intensity or duration. This highlights the critical importance of the HIRT definition (≥75% 1RM): only near-maximal loads appear sufficient to induce the neural adaptations (*e.g.*, increased rate of force development) necessary to improve economy without inducing unwanted hypertrophy, which could otherwise be detrimental to the power-to-weight ratio in endurance sports. Overall, the endurance-related findings should therefore be interpreted more conservatively than the strength outcomes, particularly when exceptionally large TTE changes are used to characterize adaptation.

## Limitations

This systematic review has several strengths, including its strict focus on elite populations and the rigorous quality assessment (PEDro scores ≥5) of the included studies. However, limitations must be acknowledged to contextualize the findings.

Firstly, despite the broad search strategy, the final selection of studies predominantly featured male participants. Only a few studies included elite female cohorts (water polo (Veliz et al., 2015 and duathlon (Vikmoen et al., 2017)), limiting the generalizability of findings to female athletes, particularly given potential sex-based differences in neuromuscular fatigue and recovery kinetics. Secondly, the scope of sporting disciplines was relatively narrow and unevenly distributed across the evidence base. Although the included studies covered several sport categories, endurance-oriented disciplines—particularly cross-country skiing and cycling—were disproportionately represented, with Scandinavian cohorts forming a large proportion of this subgroup. By contrast, conclusions for team sports (*e.g.*, soccer) and aquatic sports (*e.g.*, water polo) were based on only a small number of studies, while evidence for skill-dominant technical sports and combat sports was absent. Therefore, cross-sport generalization should be made cautiously, and the transferability of the present findings beyond the most frequently represented endurance contexts remains limited. Thirdly, the sample sizes in the included studies were generally small (median *n* = 19). While this reduces statistical power, it is a common characteristic of high-performance research, where access to elite-level populations is logistically restricted. Consequently, effect size estimates should be interpreted with caution. In addition, the present review did not apply the GRADE framework to assess outcome-level certainty of evidence. Therefore, although study-level methodological quality was evaluated using PEDro, the overall certainty of evidence across outcome domains should be interpreted cautiously and is likely to range from low to moderate, given the generally small sample sizes and the limited number of studies available for several outcomes. Fourthly, a potential language bias exists, as the search was restricted to English-language journals. This may have excluded relevant data published in other languages (*e.g.*, Chinese or Russian coaching literature). In addition, formal between-group effect sizes could not be consistently computed, as several studies lacked complete group means and dispersion data; the synthesis therefore reflects the direction of evidence rather than pooled between-group effect estimates. Finally, substantial heterogeneity in training protocols (*e.g.*, definitions of “high intensity” ranging from 75% to 90% 1RM) and testing modalities (*e.g.*, differing sprint distances) precluded a robust meta-analysis. Future research should aim to standardize testing protocols to facilitate quantitative aggregation of data.

## Conclusions

In conclusion, the available evidence suggests that High-Intensity Resistance Training (HIRT, ≥75% 1RM), when integrated into concurrent training programs, can effectively improve several aspects of athletic performance in elite athletes. Across the included studies, HIRT was associated with improvements in maximal strength (approximately 10–45%) and neuromuscular power (generally ∼5–20%) across diverse sporting disciplines, with no clear evidence of detrimental effects on aerobic-capacity-related outcomes. Endurance-related outcomes, including movement economy and fatigue resistance (*e.g.*, time to exhaustion), also generally favored HIRT, although these findings should be interpreted as the additive effects of HIRT within broader concurrent training programs rather than as the isolated effect of HIRT alone. Because all included studies used concurrent training designs, the independent effect of HIRT cannot be fully disentangled from the accompanying sport-specific training stimulus. In addition, as the present review did not apply the GRADE framework and given the generally small sample sizes and limited number of studies within several outcome domains, the overall certainty of evidence should be interpreted as likely low to moderate. Evidence for preplanned change-of-direction (COD) speed remains particularly preliminary, as it is currently derived from a single study in elite male soccer players and therefore requires replication before broader conclusions can be drawn. Adverse events were not systematically reported across the included studies; therefore, conclusions regarding safety should remain cautious.

Practical applications: For coaches and sport scientists, the available evidence supports the integration of HIRT components within concurrent training programs in elite sport settings. Across the included studies, interventions typically ranged from 5 to 18 weeks in duration and were performed one to three times per week, although considerable variation existed across sport contexts and training designs. Concurrent training programs incorporating HIRT components at 2–3 sessions per week over approximately 8–16 weeks appeared effective in several elite endurance and team-sport populations, but this should not be interpreted as a universal prescription. However, given the high-performance baseline of elite athletes, protocols must be carefully individualized to ensure adequate recovery and minimize interference with technical training. These practical implications should be interpreted in light of the likely low-to-moderate certainty of evidence and the fact that the included studies evaluated HIRT within concurrent training environments.

Future directions: To enhance the translational value of these findings, future research should prioritize underrepresented populations, particularly elite female athletes and those engaged in skill-dominant technical sports (*e.g.*, racket sports). Because the current evidence for preplanned COD speed is limited to a single study in male soccer players, future research should examine whether similar effects can be replicated in elite female and male athletes from multidirectional sports such as soccer, basketball, handball, rugby, and racket sports. Such studies should clearly distinguish between preplanned COD speed and true perceptual-cognitive agility and should use both closed COD tests and ecologically valid reactive agility assessments. Future studies should also attempt to isolate the specific contribution of HIRT within concurrent training designs and, where possible, incorporate outcome-level certainty assessment to strengthen evidence interpretation.

##  Supplemental Information

10.7717/peerj.21807/supp-1 Supplemental Information 1 PRISMA Checklist

10.7717/peerj.21807/supp-2 Supplemental Information 2 Extracted raw data from the included studies

10.7717/peerj.21807/supp-3 Supplemental Information 3 Full search strategies for all databases and search engines

10.7717/peerj.21807/supp-4 Supplemental Information 4 Codebook

## Abbreviations

1RM: One Repetition Maximum
BMI: Body Mass Index
CMJ: Countermovement Jump
COD: Change of Direction
DP: Double Poling
HIRT: High-Intensity Resistance Training
MVC: Maximum Voluntary Contraction
PF: Peak Force
PPO: Peak Power Output
RFD: Rate of Force Development
ST: Sprint Time
TTE: Time to Exhaustion
TV: Throwing Velocity
VO_2_ max: Maximal Oxygen Uptake
VO_2_ peak: Peak Oxygen Uptake
YYIRT: Yo-Yo Intermittent Recovery Test

## Additional Information and Declarations

### Data Availability

The following information was supplied regarding data availability: This is a Systematic Review/Meta-Analysis.
