# Supplementary material for: Effects of high-intensity resistance training within concurrent training programs on athletic performance in elite athletes: a systematic review
Source: PeerJ. 2026 Oct 9;14:e21807. doi: 10.7717/peerj.21807 (PMC13657067; doi:10.7717/peerj.21807)
Supplement: Supplementary file 2 [file peerj-14-21807-s003.docx]

**Supplementary Appendix 1. Full Search strategies for all databases and search engines (Searched on 27 December 2024)**

| Databases | search strategy | Results |
| --- | --- | --- |
| PubMed | ("high-intensity resistance training" OR "strength training" OR "resistance exercise") AND ("elite athletes" OR "professional athletes" OR "competitive athletes") AND ("sports performance" OR "athletic performance" OR "strength" OR "explosive power" OR "agility") | 87 |
| Web of Science | TS=("high-intensity resistance training" OR "strength training" OR "resistance exercise") AND TS=("elite athletes" OR "professional athletes" OR "competitive athletes") AND TS=("sports performance" OR "athletic performance" OR "strength" OR "explosive power" OR "agility") | 5 |
| SCOPUS | TITLE-ABS-KEY(("high-intensity resistance training" OR "strength training" OR "resistance exercise")) AND TITLE-ABS-KEY(("elite athletes" OR "professional athletes" OR "competitive athletes")) AND TITLE-ABS-KEY(("sports performance" OR "athletic performance" OR "strength" OR "explosive power" OR "agility")) | 147 |
| SPORTDiscus | TI ("high-intensity resistance training" OR "strength training" OR "resistance exercise") AND TX ("elite athletes" OR "professional athletes" OR "competitive athletes") AND TX ("sports performance" OR "athletic performance" OR "strength" OR "explosive power" OR "agility") | 164 |
| Google Scholar | "high-intensity resistance training" OR "strength training" OR "resistance exercise" AND "elite athletes" OR "professional athletes" OR "competitive athletes" AND "sports performance" OR "athletic performance" OR "strength" OR "explosive power" OR "agility" | 1140 |
| Total |  | 1543 |
